# Place du vaccin polio inactivé dans le Programme élargi de vaccination

**DOI:** 10.48327/mtsi.v3i2.2023.380

**Published:** 2023-06-02

**Authors:** Pierre SALIOU

**Affiliations:** Président honoraire de la SFMTSI, Société francophone de médecine tropicale et santé internationale (ancienne SPE), Hôpital Pitié-Salpêtrière, Pavillon Laveran, 47-83 Boulevard de l'Hôpital, 75651 Paris cedex 13, France; Voir l'article de Martin SCHLUMBERGER Augmentation de l'efficience d'un PEV en stratégie mobile utilisant le vaccin polio injectable en Afrique. Med Trop Sante Int. 2023(3):2:mtsi.v3i2.2023.344

**Keywords:** Poliomyélite, Éradication, PEV, Vaccin polio inactivé, Vaccin polio oral, Efficience, Afrique, Poliomyelitis, Eradication, EPI, Killed polio vaccine, Live polio vaccine, Efficiency, Africa

## Abstract

Il peut paraître surprenant que le Comité de rédaction de *Médecine Tropicale et Santé Internationale (MTSI)* accepte de publier l'article intitulé « Augmentation de l'efficience d'un PEV en stratégie mobile utilisant le vaccin polio inactivé en Afrique^01^01Martin SCHLUMBERGER Augmentation de l'efficience d'un PEV en stratégie mobile utilisant le vaccin polio injectable en Afrique. Med Trop Sante Int. 2023(3):2:mtsi.v3i2.2023.344 », 35 ans après la fin du travail effectué en 1988. Je livre brièvement ici la justification de cette décision.

Martin SCHLUMBERGER Augmentation de l'efficience d'un PEV en stratégie mobile utilisant le vaccin polio injectable en Afrique. Med Trop Sante Int. 2023(3):2:mtsi.v3i2.2023.344

## Abstract (en)

It may seem surprising that the Editorial Board of *Médecine Tropicale et Santé Internationale (MTSI)* would agree to publish the article “Increasing the efficiency of a mobile EPI strategy using injectable polio vaccine in Africa” 35 years after the work was completed in 1988. I briefly outline the rationale for this decision here.

Il peut paraître surprenant que le Comité de rédaction de *Médecine Tropicale et Santé Internationale (MTSI)* accepte de publier l'article intitulé « Augmentation de l'efficience d'un PEV en stratégie mobile utilisant le vaccin polio inactivé en Afrique », 35 ans après la fin du travail effectué en 1988. Je livre brièvement ci-dessous la justification de cette décision.

Dans un avant-propos accompagnant son manuscrit, l'auteur, Martin Schlumberger, écrit que l'UNICEF, partenaire de l’étude, avait en son temps opposé un veto formel à cette publication qui démontrait la supériorité économique avec une efficacité clinique quasiment identique du vaccin poliomyélitique inactivé injectable (VPI) par rapport au vaccin poliomyélitique vivant atténué oral (VPO), dans le Programme élargi de vaccination (PEV).

Il faut bien avoir à l'esprit qu’à la fin des années 1980, lorsque l'OMS s'est lancée dans l'Initiative mondiale pour l’éradication de la poliomyélite (IMEP), l'UNICEF était le principal bailleur de fonds du VPO qui a été, pendant de très nombreuses années, le « vaccin de choix » pour l’éradication de la poliomyélite. Le veto de l'UNICEF peut s'expliquer par le fait qu'il ne fallait pas perturber les campagnes de vaccination par le VPO qui débutaient dans le cadre de l’éradication. J'ai personnellement travaillé à la fin des années 1970 sur l'introduction d'un nouveau vaccin poliomyélitique inactivé concentré développé par Jonas Salk, dans le cadre du Programme élargi de vaccination. C'est d'ailleurs le vaccin utilisé par M. Schlumberger dans son travail. À l’époque, pour l'OMS, la supériorité du VPO par rapport au VPI pour les PEV et l’éradication ne se discutait pas pour diverses raisons (coût du vaccin inférieur, vaccination de l'entourage par les virus vaccinaux excrétés…). C’était quasiment dogmatique! Pourtant, l'efficacité de ce nouveau vaccin inactivé a été démontrée. Il a fini par s'imposer quand sont apparus les poliovirus dérivés du vaccin oral, à l'origine d’épidémies de paralysies flasques dans le début des années 2000.

La date de l’éradication de la poliomyélite est régulièrement retardée, en particulier du fait de l'apparition de ces poliovirus dérivés du vaccin. Aujourd'hui, l'OMS espère atteindre cet objectif fin 2026… Depuis plusieurs années déjà, le VPI est inclus dans le PAMV, certes en association avec le VPO pour l'instant^1^1Le Plan d'action mondial pour les vaccins (PAMV) était la stratégie mondiale de vaccination de la « Décennie de la vaccination » (2011-2020). Il a remplacé le Programme élargi de vaccination et se poursuit depuis 2021 sous le nom de Programme pour la vaccination à l'horizon 2030 [1, 2].. Mais quand l’éradication sera atteinte, le VPO devra être supprimé de tous les programmes afin de ne plus « inonder » le monde de virus vaccinaux dont le risque est de muter et de se recombiner en virus pathogènes. En revanche, le VPI devra continuer à être intégré dans les programmes de vaccination, sans doute pendant longtemps, jusqu’à la certification de l’éradication.

En somme, les trois décennies qui se sont écoulées depuis la fin du travail de M. Schlumberger ont montré que son article aurait mérité d’être publié dès que le travail fut terminé. En effet, depuis, le VPI a pris sa revanche sur le VPO ! Il convient de rappeler ici que même si les résultats d'une étude ne vont pas dans le sens attendu, la rigueur scientifique impose de les publier. C'est, je pense, quasiment à titre d'exemple que le Comité de rédaction de *MTSI* a accepté cette publication. J'ai tenté d'expliquer pourquoi.

## Liens D'intérêts

L'auteur ne déclare aucun lien d'intérêt
